# Enzymatic Conversion of Mixed Neem (*Azadirachta indica*) and Nile Tilapia (*Oreochromis niloticus*) Oils into Biolubricants: A Green Biocatalytic Approach

**DOI:** 10.1021/acsomega.4c11707

**Published:** 2025-05-20

**Authors:** Francisco Simão Neto, Patrick da Silva Sousa, Rafael Leandro Fernandes Melo, Antônio Luthierre Gama Cavalcante, Paulo Gonçalves de Sousa Junior, Sara Jessica Marciano, Diego Lomonaco, Raniere Dantas Valença, Frederico Ribeiro do Carmo, Marcos Carlos de Mattos, Maria Alexsandra de Sousa Rios, Paulo Roni de Souza, Aluísio Marques da Fonseca, Ada Sanders Lopes, José Cleiton Sousa dos Santos

**Affiliations:** † Departamento de Engenharia Química, 28121 Universidade Federal do Ceará, Fortaleza, CE 60455-760, Brasil; ‡ Departamento de Engenharia Metalúrgica e de Materiais, Universidade Federal do Ceará, Campus do Pici, Fortaleza, CE 60714-903, Brasil; § Departamento de Química Orgânica e Inorgânica, Universidade Federal do Ceará, Fortaleza, CE 60455-760, Brasil; ∥ 74384 Universidade Federal Rural do Semi-Árido, Mossoró, RN 52625-900, Brasil; ⊥ Núcleo de Pesquisa em Economia de Baixo Carbono, Centro de Engenharias, Universidade Federal Rural do Semi-Árido, Mossoró, RN 52625-900, Brasil; # Departamento de Química Analítica e Físico-química, Universidade Federal do Ceará, Fortaleza, CE 60455-760, Brasil; ∇ Departamento de Engenharia Mecânica, Universidade Federal do Ceará, Fortaleza, CE 60455-760, Brasil; $ Instituto de Engenharia e Desenvolvimento Sustentável, 245069 Universidade da Integração Internacional da Lusofonia Afro-Brasileira, Campus das Auroras, Redenção, CE 62790-970, Brasil

## Abstract

This study focused on optimizing the enzymatic synthesis of biolubricants using a 20% blend of Nile tilapia (*Oreochromis niloticus*) and neem (*Azadirachta indica*) oils catalyzed by Eversa Transform 2.0. The optimal conditions—the molar ratio of 1:5 (mol/mol), 10% biocatalyst load, and a reaction time of 48 h—achieved a predicted ester conversion of 97%, with experimental validation confirming 92.80 ± 0.03%. Molecular docking demonstrated stable complexes, particularly with decanoic and linoleic acids, showing Root Mean Square Deviation (RMSD) values below 2.0 Å, indicating minimal conformational changes. Gas chromatography and mass spectrometry (GC-MS) analysis identified key esters like 2-ethylhexyl and octyl, which enhance thermal stability, lubricity, and oxidation resistance. Fourier Transform Infrared Spectroscopy (FT-IR) analysis confirmed effective ester formation, corroborating experimental results. Viscosity (2.4511 mm^2^/s) and density (0.84259 g/cm^3^) values aligned with ISO VG 3 (International Organization for Standardization Viscosity Grade) standards, suitable for low-load and low-speed applications.

## Graphical abstract

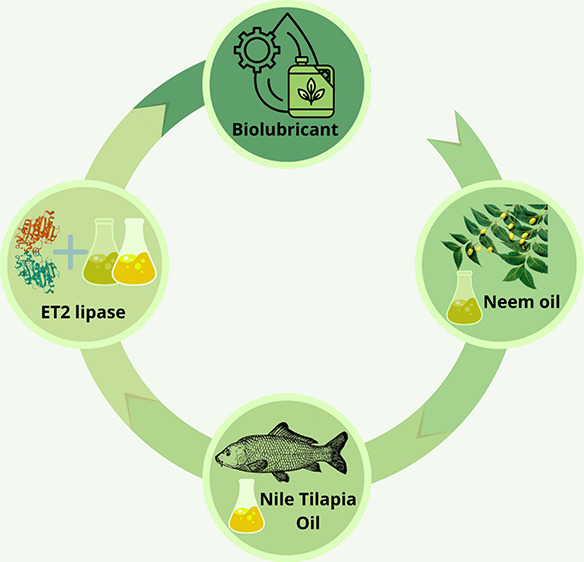

## Introduction

In 2004, the worldwide lubricant market demand reached 37.4 million metric tons, with an annual growth rate of 2% since then, where the monetary yield for 2025 is expected to be 166.25 billion dollars.^1,2^ Lubricants are a prime requirement for the smooth operation of machinery, as all machines require lubrication, and 50% of fuel energy is lost to overcome frictional losses in an internal combustion engine.^3–5^ The lubricant obtained from petroleum presents problems related to disposal and environmental damage as it contains non-degradable and toxic agents.^6,7^ One liter of mineral lubricating oil can contaminate one million liters of water.^8,9^ Therefore, researchers have directed their work towards developing cleaner and more sustainable processes.^10,11^ Biodegradable products are, consequently, substitutes for petroleum-based products due to their biodegradability and non-toxicity.

In addition, they are environmentally friendly, are clean, and use low-cost raw materials.^12,13^ While biolubricants are becoming more cost-effective, mineral oils remain cheaper and more readily available on a large scale, making them the preferred choice for many industries.^9,12,14^ Biolubricants share only 4–5% of the industrial applications and engines in eight markets. It is worth noting that vegetable oils, edible or non-edible, can be used as raw material to produce biolubricants.^6,15^ Oxidation of vegetable oil is a disadvantage for its use as a lubricant.^16^ Vegetable triglycerides are good for limiting lubrication, as the amount of unsaturation in vegetable oil restricts its use and suitability as a mineral oil-derived lubricant.^17^ Therefore, work has been developed with oil blends to reduce disadvantages and maximize physical properties relevant to bioproduct commercialization.^18–20^

The neem tree (*Azadirachta indica*) has been investigated as a raw material for applications in the bioenergy sector.^21–23^ It is a plant native to India that has adapted to the climate of Northeast Brazil, enabling its cultivation and use in the synthesis of biofuels.^24,25^ Research indicates that the oil extracted from seeds can be used in repellents, pesticides, and even personal hygiene items.^26,27^ In addition, a study on the extraction of neem oil indicated that 45% of the mass of its seed is made up of oil and presented the following composition: oleic acid (50–60%), palmitic acid (13–15%), stearic acid (14–19%), linoleic acid (8–16%) and arachidic acid (1–3%) demonstrating potential for proper application in the production of biodiesel and biolubricants.^27^

The importance of fishing is justified by the increase in fish consumption, which in 1961 was approximately 9.0 kg per capita, with a significant increase in 2015 to 20.2 kg per capita (FAO, 2020).^28^ The global fish market was valued in 2019 at 1,905.77 million dollars and is expected to reach 2,844.12 million dollars in 2027.^29^ The consequence is fish processing waste equivalent to 250 billion tons, which can increase annually and be used to obtain value-added products, such as biolubricants.^30^ It is essential to highlight that fish oil is found in different parts and various quantities, such as the head, viscera, and tail.^31^ Nile tilapia (*Oreochromis niloticus*) oil contains fatty acid triglycerides, and its composition differs from vegetable oils due to the presence of a large amount of long-chain PUFAs.^32^

The synthesis of biolubricants occurs mainly through chemical catalysis using organic substrates that can be modified through the esterification and transesterification routes.^33^ However, given the importance of adopting the principles of green chemistry in industrial-process synthesis the enzymatic route was introduced.^34–36^ Among the advantages are mild temperatures for carrying out the reaction and elimination of subsequent purification steps.^37,38^ Enzymes are biological catalysts with a specific catalytic activity that accelerates various reactions.^39,40^ Its use in industrial production chains provides advantages over traditional chemical catalysis due to its sustainability and efficiency.^41^

For industrial viability, it is essential to make biocatalysts cheaper, ensuring efficiency.^42–44^ An example is Eversa Transform 2.0 (ET2), commercially named and is the *Thermomyces Lanuginosus* lipase obtained from a genetically modified strain of *Aspergillus oryzae*.^45,46^ It contains 269 amino acid residues, a molecular weight of 31.5 kDa, 9100 IU/mL activity, and a production cost equivalent to 15 dollars per kilo.^47,48^ Its catalytic site is composed of the catalytic triad: serine (153), histidine (268) and aspartic acid (206).^49–51^ Novozymes possesses the patent for manufacturing a more efficient biocatalyst than the original enzyme, yet few scholarly papers document its usage in biotechnological processes.^45^

Therefore, mixtures of vegetable oil obtained from the neem plant (*Azadirachta indica*) and residual fish oil from Nile tilapia (*Oreochromis niloticus*) will be used to evaluate the biocatalytic production of biolubricants. The lipase used in this process will be ET2. The objective is to assess the reaction parameters that significantly affect the optimization of operational conditions and eventually maximize bioproduct conversion. Therefore, the present study aims to collaborate in synthesizing and developing sustainable biochemical processes that mitigate pollution and environmental damage.

## Methodology

### Biocatalyst and Chemical Reagents

Commercial lipase Eversa transform 2.0 (ET2) from *Thermomyces lanuginosus* (*Tl*L) (CAS: 9001-62-1; COD: SAE0065; Sigma-Aldrich Brazil Ltda), produced by Novozymes through the submerged fermentation of a genetically modified strain of *Aspergillus oryzae* and lipase from *Thermomyces lanuginosus* (*Tl*L). Chemical reagents used, such as 2-ethylhexan-1-ol alcohol (>99% wt.; CAS: 104-76-7; COD. 538051; Sigma-Aldrich Brazil Ltda), were analytical grade from Synth and Vetec. Using the Taguchi method, Statistica 10 software (Statsoft) was used for experimental design.

### Enzymatic Activity Assay

This study obtained esters from neem and Nile tilapia oils using enzymatic hydro esterification in two phases. Initially, the oil was hydrolyzed to form free fatty acids (FFA) using the TlL biocatalyst. The oil–water solution (1:1) was heated to 40 °C, adding 0.4% of biocatalyst. After 4 h of agitation at 40 °C, the solution was transferred to a separation funnel. FFA washed three times with water at 60 °C, the aqueous phase was removed, and FFA was heated at 80 °C for 10 min. Then, the mixture was filtered using filter paper and 20% w/v anhydrous sodium, previously dried at 250 °C for 4 h.

Following the experimental design evaluations, the activity assay was carried out with reaction mixtures containing different proportions of reagents at different temperatures and for various time intervals. 2 mL flasks were used to carry out biochemical reactions under controlled incubator temperature and maintain an orbital agitation of 200 rpm. In these reactions, a calculation was made based on the density and molarity of the acids and alcohol so that it was possible to quantify the substrates by volume while ensuring that the molarity was respected. Volume favors the quantification of free enzymes used in the tests. Thus, the volume of oil was kept constant, while the volume of alcohol was altered by the planning from 1:1 to 1:9. Similarly, the amount of biocatalyst was increased as the design was applied, ranging from 5 to 15% biocatalyst, the amount of oil used. After completing the esterification reaction, the samples were analyzed in triplicate in two Erlenmeyer flasks with 0.2 grams of the sample, 5 mL of standard ethyl alcohol and three drops of phenolphthalein as an indicator. Each sample was titrated with 0.1 M NaOH solution until the color changed to a subtle pink. After titration, the total volume consumed was used in eq 1 to obtain the acidity index (AI).

1 $$\mathrm{AI}=\frac{{\mathrm{MW}}_{\mathrm{NaOH}}\cdot M_{\mathrm{NaOH}}\cdot f\cdot V_{\mathrm{NaOH}}}{m}$$

In eq 1, MW_NaOH_ is the molecular mass of NaOH, M_NaOH_ is the molarity of the NaOH solution used in the titration, f is the correction factor determined by NaOH standardization, V_NaOH_ is the total volume of NaOH consumed during the titration, and m is the mass of the analyzed sample.^52^ The conversion (X) of free fatty acids from the blend produced from neem and Nile tilapia oils into the biolubricants is then given by eq 2, where AI_0_ represents the initial acidity value due to the starting concentration of acid in the sample. AI represents the final acidity value, equivalent to the remaining acid in the solution, not consumed during the enzymatic reaction.

2 $$X\left(\%\right)=\frac{{\mathrm{AI}}_{0}-\mathrm{AI}}{{\mathrm{AI}}_{0}}\times 100$$

### Experimental Design and Statistical Analysis

An experimental design based on the Taguchi method was used with a standard L9 orthogonal matrix (the “L” and “9” represent the Latin square and the number of experiments, respectively) to distribute four factors in three levels to maximize the conversion.^53,54^ The four independent factors: the molar ratio between organic acid and alcohol (MR), the biocatalyst load (Cat), the reaction time (t), and blend (BL) between Nile tilapia oil (reference oil) and neem oil, as well as their corresponding levels are shown in Table 1.

**Table 1:** Determination of Experimental Procedure Levels and Range of Independent Parameters

**Level**	**MR** **(mol/mol)**	**Cat (%)**	**BL (%)**	**t (h)**
Level 1 (L1)	1:1	5	20	48
Level 2 (L2)	1:5	10	50	72
Level 3 (L3)	1:9	15	80	96

The load of the biocatalyst was calculated from the volume of the reaction after calculating the molar ratio between the mixture of acids and alcohol, starting from a ratio of 1 mol of acids and 1 mol of alcohol (L1) and going up to a ratio of 1 mol of acids and 9 mol of alcohol (L3). The values of signal-to-noise ratios (S/N) corresponding to the conversions were calculated using the characteristics of the “bigger is better” function since the objective of this study is to maximize the response (biolubricant ester production).^55^ In the Taguchi method, the S/N ratio measures quality characteristics and deviation from the desired value. Thus, using S/N ratio to analyze the results reduces the system’s sensitivity to sources of variation, resulting in a good performance. The value of the S/N ratio for each experiment was calculated according to eq 3.

3 $$S/N=-10\log\left(\frac{1}{n}\overset{n}{\underset{i=1}{\sum }}\frac{1}{{y_{i}}^{2}}\right)$$

In eq 3, y is the fatty acid conversion for the corresponding sample, i is the number of replicates, and n is the number of responses for the combination of factor levels in any given parametric combination. The predicted signal-to-noise ( $\overset{\sim }{S/N}$ ) ratio under optimal conditions for obtaining the maximum conversion was estimated by eq 4.

4 $$\overset{\sim }{S/N}=\frac{\mathrm{S̅}}{N}+\overset{n}{\underset{i=1}{\sum }}\left(\frac{S}{N_{j}}-\frac{\mathrm{S̅}}{N}\right)$$

In eq 4, $\frac{\mathrm{S̅}}{N}$ is the arithmetic mean of all S/N ratios, S/N_j_ is the S/N ratio at the sweet spot for each factor, and n is the number of factors significantly affecting the process.

### Quantification and Characterization of the Products

The one-dimensional spectra of Hydrogen Nuclear Magnetic Resonance (^1^H NMR) and Carbon-13 Nuclear Magnetic Resonance (^13^C NMR) were obtained using a Bruker Advance DRX-500 spectrometer, provided by the Northeastern Center for Application and Use of Nuclear Magnetic Resonance at the Federal University of Ceará (CENAUREMN-UFC). Measurements were conducted at a hydrogen frequency of 500 MHz and a carbon frequency of 125 MHz to identify the hydrogen and carbon peaks corresponding to the structure of octyl oleate. Deuterated chloroform (CDCl_3_) was used as the solvent to dissolve the samples examined in 5 mm tubes.^56^

To determine the ester (C) content of the biolubricant samples produced, an analysis was carried out by gas chromatography with coupled mass spectrometry (GC/MS), following the methodology adapted from EN 14103 standard.^57^ The methodology consists of using approximately 50 mg of the biolubricant sample. This biolubricant was mixed in a 2 mL vial with a solution of methyl nonadecanoate (10 mg/mL). This mixture was then injected (1 μL) into a SHIMADZU QP-2010 ULTRA chromatograph equipped with a (5%-phenyl)-methylpolysiloxane (DB-5) capillary column (30 m × 0.25 mm × 0.25 μm film thickness), using helium as carrier gas in splitless mode.

FT-IR spectra were recorded using a Perkin Elmer FT-IR/NIR FRONTIER spectrophotometer equipped with an attenuated total reflectance (ATR) accessory featuring a ZnSe, with a resolution of 4 cm^–1^, using the arithmetic mean of 32 scans in the wavenumber range of 4000–500 cm^–1^.

Viscosity and density were determined in an Anton Paar SVM 1101 digital oscillation U-tube apparatus. This device is entirely ASTM D4052 and ISO 12185 compliant density measurement. The sample volume of 3.5–8 mL was injected into the device to measure the properties of produced liquids. The measure of viscosity was performed in a cell containing a tube filled with a sample rotating at a constant speed. The density was determined by a densimeter using the U-tube principle. Both measurements were done simultaneously. The density measurement has an uncertainty of 0.00005 g·cm^–3^, and the viscosity measurement has an error of 0.2%. The measurements were performed at 40 °C, followed by ASTM D2270-24.

### Study *in Silico*

The molecules of a blend of the Nile tilapia oil (*Oreochromis niloticus*) and neem (*Azadirachta indica*) using ET2 were created by the Chem3D software^58^ (Figure 1). In the auto-optimization settings, the force field has been applied MMFF94,^59^ to generate bioactive conformations by minimization of randomly generated conformers, with Steepest Descente algorithm,^60^ Stepper Update 4,^61^ and the AVOGADRO software.^62^ The file with ligand was converted to the corresponding format (.pdbqt) by adding ionization and tautomeric states at pH 7.4 using OpenBabel ver. 3.0.0 software.^63^

**Figure 1:**
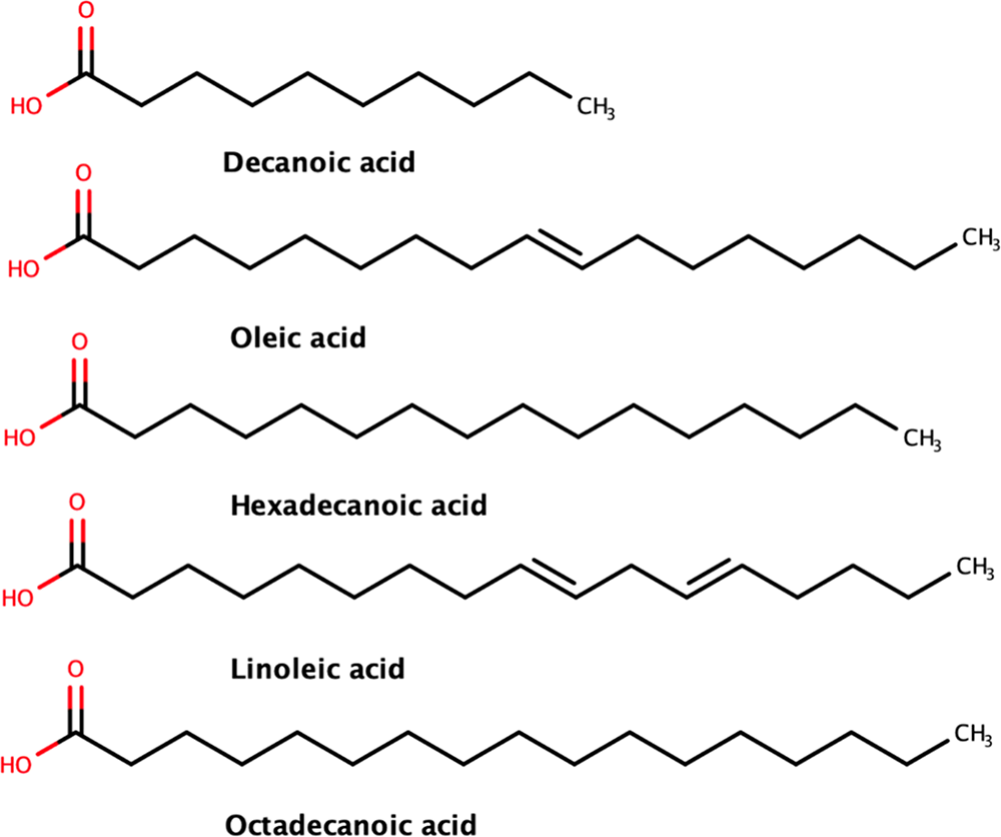
Structures in 2D of the blend of the residual oil of Nile tilapia (*Oreochromis niloticus*) and neem (*Azadirachta indica*).

The receptor under study was the structure of the lipase ET2 that was modelled in the following literature procedures,^64,65^ whose crystalline structure was obtained by complex X-ray diffraction. The interfering residues, water molecules, and the synthetic inhibitor were removed to make molecular docking possible. Polar hydrogens were added to the ligands, separately, and the protein. This technique searches inside potential virtual ligand databases for a given protein target. The software used was Autodock tools.^66^

Molecular docking was performed by AutoDock Vina^67^ employing 3-way multithreading, Lamarkian Genetic was performed. The parameters used for coupling the ET2 main protease complex are shown in the Supporting Information. Thus, to validate the simulation’s performance and quantify the dockings’ qualitythe RMSD (root mean square deviation) scoring criterion was adopted, which suggests that a successful docking exhibits an RMSD value of ≤2.0 Å.^68^ The simulation data with the main receptor–ligand interactions were visualized by Discovery Studio software.^69^ Molecular dynamics (MD) simulations were performed with the NAMD program.^70^ The parameters used for the MD simulations are shown in the Supporting Information.

Results are expressed as mean values ± standard error of the mean. After confirming the normal distribution and homogeneity of the data, differences between the groups were subjected to analysis of variance (one-way ANOVA) and two-way ANOVA in experiments with antagonists,^71^ followed by Tukey’s test.^72^ All analyses were performed using GraphPad Prism v.8.0 software. The level of statistical significance was set at 5% (p < 0.05).

## Results and Discussion

### Process Optimization by the Taguchi Method

Table 2 presents a Taguchi L9 experimental design for the mixture of neem oil (*Azadirachta indica*) and Nile tilapia oil (*Oreochromis niloticus*), intending to investigate the efficiency of esterification to produce sustainable biolubricants using ET2 lipase.

**Table 2:** Experimental Design of the Taguchi L9 Plan^a^

**Experiment**	**MR** **(mol/mol)**	**Cat (%)**	**BL (%)**	**t (h)**	**X (%)**	**S/N**
1	1:1	5	20	48	89.1 ± 0.1	38.99
2	1:1	10	50	72	86.0 ± 0.1	38.69
3	1:1	15	80	96	68.9 ± 0.1	36.76
4	1:5	5	50	96	81.0 ± 0.6	38.17
5	1:5	10	80	48	73.6 ± 0.3	37.33
6	1:5	15	20	72	78.6 ± 0.6	37.90
7	1:9	5	80	72	53.3 ± 0.2	34.53
8	1:9	10	20	96	82.0 ± 0.5	38.27
9	1:9	15	50	48	63.6 ± 0.6	36.07

a Every point was performed in triplicate to ensure the error was within the standard limits. **MR**: Molar ratio; **Cat**: Percentage of biocatalyst; **BL**: Mixture of oils, where Nile tilapia oil is the reference; **t**: time; **X**: Conversion; **S/N**: Signal-to-noise ratio.

The molar ratio between the reactants varied from 1:1 to 1:9. It can observed that the highest esterification percentages were obtained with a molar ratio of 1:1, with 89.1 and 86.0%, respectively, in experiments 1 and 2. The literature indicates that the molar ratio is a critical factor in esterification, as it determines the relative concentration of the reactants.^73,74^ Very high or very low molar ratios can negatively affect reaction efficiency due to the imbalance in the availability of substrates for the enzyme.^75,76^ In general, moderate molar ratios tend to maximize conversion, so the behavior presented in this study aligns with the literature. In addition, experiments 7 and 9 had molar ratios of 1:9 and showed the lowest conversions, 53.3 and 63.6%, respectively.

The biocatalyst load varied from 5% to 15%. A 5% load in experiment 1 resulted in the highest esterification percentage (89.1%), while a 15% load in experiment 6 resulted in 78.6%, so increasing the biocatalyst load does not necessarily lead to an increase in conversion. The literature on esterification with lipases suggests that increasing the amount of biocatalyst generally improves the reaction rate to a point where more enzymes doot bring additional benefits due to saturation of the active sites.^76,77^ Biocatalyst loads of 5% and 10% showed good yields, while 15% did not provide significant additional advantages. As a form of validation, by keeping the biocatalyst load at 5% and varying the molar ratio from 1:1 to 1:9 in experiments 1, 4 and 7, respectively, at 1:9 there is a considerable reduction in conversion, going from 89.1% in experiment 1 to 53.3% in experiment 7. These results corroborate what was discussed in the previous paragraph about molar ratios.

The blend of oil varied from 20% to 80%. It has been noted that the blend of 80% Nile tilapia oil (experiment 3) resulted in a low esterification percentage (68.9%). In comparison, the combination of 50% of each oil (experiment 2) resulted in a high conversion (86.0%). The proportion of oils can affect the fatty acid composition and, consequently, the reactivity in esterification.^78–80^ Studies indicate that balanced combinations of different oils can optimize enzymatic activity and ester production.

The reaction time varied from 48 h to 96 h. The results show that longer times do not always correlate with higher efficiency. Experiment 8, with 96h of reaction, resulted in a high conversion (82.0%), while experiment 3, simultaneously resulted in a low conversion (68.9%). This suggests that the reaction time should be optimized with other variables. The literature also supports the need for a balance, where sufficient time is given to achieve maximum conversion without excessively prolonging the reaction, which can lead to product degradation.^76^

Experiment 7 showed a significantly lower conversion rate (53.3%) than the other experiments. This can be attributed to the specific combination of variables tested: a molar ratio of 1:9, a biocatalyst load of 5%, a combination of 80% neem and 20% tilapia, and a reaction time of 72 h. The high molar ratio may have diluted the active reactants, while the low biocatalyst load may not have sufficiently catalyzed the reaction effectively. Additionally, the combination of oils may not have been ideal for enzymatic activity. This result underscores the importance of optimizing all variables to achieve maximum esterification efficiency.^81,82^

The data analysis suggests that the combination of oils and the molar ratio is most significant in esterification efficiency. The molar ratio affects the availability of substrates, while the combination determines the number of fatty acids from each oil. The biocatalyst load and reaction time are also essential but should be optimized with the other factors.

Studies with ET2 lipase in other oils, such as soybean and palm, show it is highly efficient, with conversions above 90% under optimized conditions.^78,79,83^ Our results aligned with these findings and demonstrated the effectiveness of ET2 in different oil combinations and reaction conditions.

### Statistical Analysis

To determine the importance and influence of each parameter studied, the Taguchi method takes a reading of the signal-to-noise ratio (S/N) values. In addition, to determine the S/N values related to the biolubricant conversions in each sample analyzed, the “the higher, the better” function was applied. Table 3 shows the average S/N data for each parameter level (L1, L2 and L3) and delta values to classify each factor and thus determine its level of influence on the process. In addition, Figure 2 graphically shows the variation in the levels used for each parameter. To determine the delta values, it is necessary to subtract the S/N value of the highest level from the value of the lowest level. In both (Table 3 and Figure 2), the parameters are represented by acronyms (MR, Cat, BL, t). However, these values represent the S/N of that respective parameter and level and not their actual utilization values. Therefore, this table does not use the actual units of the parameters. It was, thus, possible to determine that the percentage of the blend and the molar ratio were the parameters that most influenced the reaction, with a delta of 2.18 and 1.86, respectively.

**Table 3:** Response to Mean S/N Ratios and Order of Variables for the Molar Ratio (MR), the Biocatalyst Load (Cat), the Combination (Com), and the Reaction Time (t)

**Factor Levels**	**MR**	**Cat**	**BL**	**t**
1	38.15	37.23	38.39	37.47
2	37.81	38.10	37.65	37.05
3	36.29	36.91	36.21	37.74
**Delta**	1.86	1.19	2.18	0.69
**Ranking**	2	3	1	4

**Figure 2:**
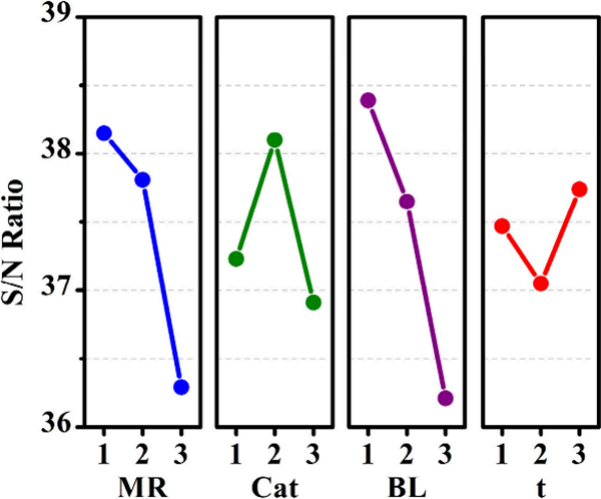
Graphic of the averages of the parameter’s variations.

Table 3 categorizes the influence of the parameters used in this study to determine the best conditions for the enzymatic synthesis of biolubricant from a mixture of Nile tilapia oil and neem oil. From the experimental results in Table 2, it was possible to highlight the influence of a mix of oils for more excellent conversion of free fatty acids into biolubricant esters. The most suitable mixture was 20% Nile tilapia oil and 80% neem oil. The analysis of the S/N responses proves this influence since the mixture (BL) ranked 1st in terms of variation. Likewise, the molar ratio between the acids and the alcohol is noteworthy, as this parameter came second in the variation of S/N. Experimentally, the molar ratio proved to be a parameter that did not require significant variation since between 1:1 and 1:5 there was already an acceptable conversion value, added to the fact that the ratio of 1:9 showed the highest conversion in the plan.

The variation and optimization of biolubricant synthesis reactions with parameters that impact the reaction yield were determined by applying the Taguchi method.^82,84–86^ In the Taguchi method, three essential instruments, such as orthogonal experimental design, S/N ratio, and ANOVA,^85,87,88^ were used to analyze and evaluate the numerical results. It is noticeable that the combination was the most impactful factor in the reaction process, obtaining a high delta and a greater degree of impact on the variation of the result. It should be noted that the higher the level of the combination, the worse the reaction result is.^88,89^ On the other hand, time is the parameter that caused the most negligible impact on the reaction process, obtaining better results in minimum reaction times according to the reaction range of the experimental design.^87,89^

In this sense, according to Gemano et al. 2022,^88^ in their study on the application of biocatalysts from ET2 lipase, it was noticed that time is the factor with the most negligible impact on the reaction yield in the production of biolubricant, obtaining better yields in reaction times at minimum levels of its experimental design.^88^ In line with Miguel et al. (2022), the combination is one of the factors that most impact the reaction process, highlighted by presenting yields above 80% with minimum combination values.^87^ These factors were relevant to this research on applying ET2 in biolubricant synthesis reactions.

The analysis of variance then showed results that corroborated the previous study. Table 4 shows the values obtained in the analysis, where the p-value is the main parameter to be considered since it is possible to determine the significance of each factor in the reaction from the p-value. To guarantee the importance of a parameter of at least 95%, the p-value of this parameter must be less than 0.05. Thus, in the present work, the blend was the most significant factor with reliability within 95% because it had a p-value of 0.037. In contrast, the molar ratio, catalyst load, and time had p-values greater than 0.05. Finally, it is worth highlighting the significant contribution of the blend in the reaction, which contributed 57.09%, followed by the molar ratio of 39.95%.

**Table 4:** Results of Analysis of Variance (ANOVA) for Parameters That Affect the Production of the Ester

**Factors**	**DF**	**SS**	**MS**	**F** **-Value**	**p** **-Value**	**Contribution (%)**
**Molar ratio**	2	338.49	169.24	6.61	0.062	39.95
**Biocatalyst**	2	25.15	12.57	0.49	0.522	2.96
**Blend**	2	483.52	241.76	9.45	0.037	57.09
**Time**	(2)	(5.40)	-	-	-	-
**Total**	6	847.16	-	-	-	100

A significance level of 0.05 indicates a 5% risk of concluding that there is a difference when there is no real difference. p-value ≤ α: Differences between some of the means are statistically significant. According to defined Melo et al. (2024),^76^ p-values were considered significant with a 95% confidence interval. In this sense, this result is once again like that in the literature.^76^ In this sense, Vieira et al. (2021)^48^ present the exact similarity in its results if its yields are better at higher temperatures. Therefore, it is possible to analyze the reaction temperature to synthesize the biolubricant for potential industrial applications.^48^

Based on the statistical analysis, the mixture was determined to be the most decisive parameter for synthesizing the biolubricant, so 2D contour surface graphs were drawn up to evaluate the interaction between the blend and the other planning parameters. Figure 3 shows the surface graphs generated to show the correlation between the parameters.

**Figure 3:**
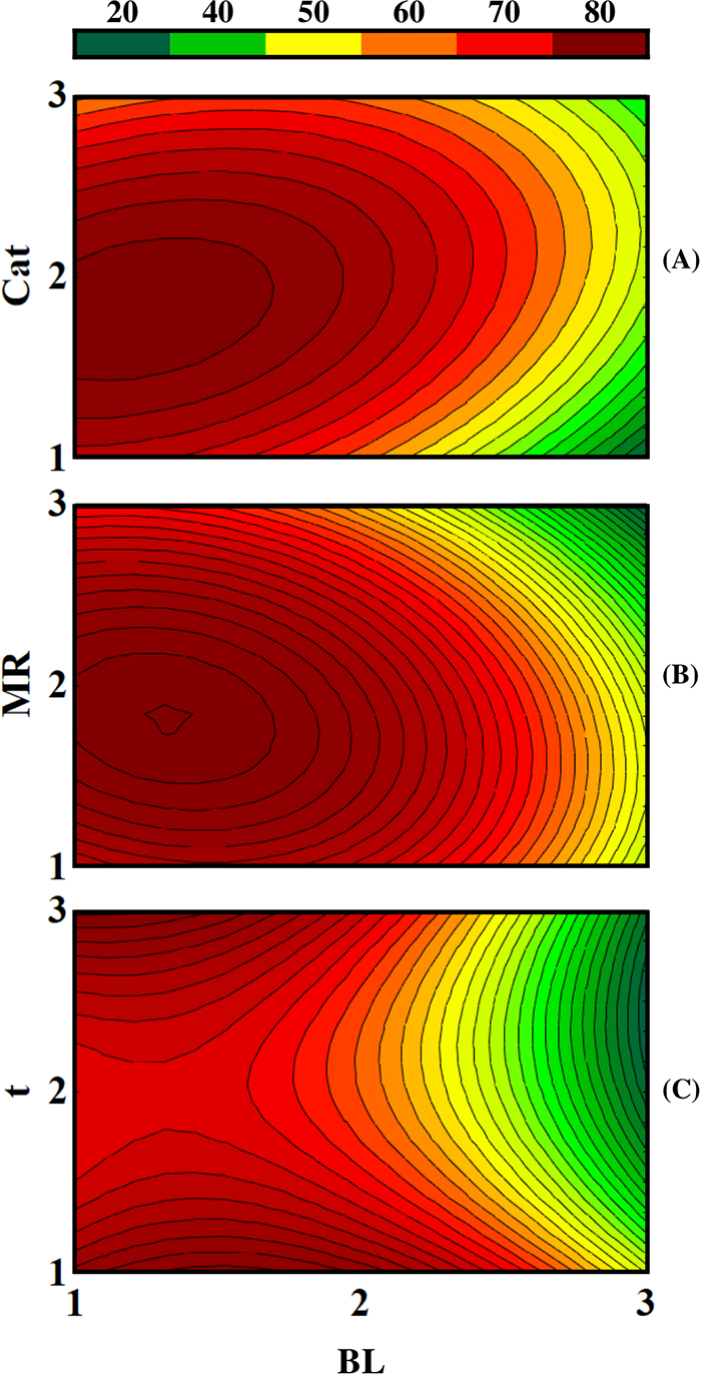
Contour surfaces to produce biolubricant. (A) Blend versus Biocatalyst. (B) Blend versus Molar Ratio. (C) Blend versus Time. The bar (legend) above the graphs refers to the produced biolubricant yield value in %.

Analysis of Figure 3A shows that the relationship between the blend and the catalyst at the highest level of variation does not increase the conversion percentage since the area of the most excellent production is located near BL level 1 and Cat level 2. Figure 3B, which relates the blend and the molar ratio, follows the same trend, where the most excellent biolubricant production area is BL level 1 and MR level 2. In Figure 3C, the scenario changes since the level where time contributes the most to conversion is level 3. Still, following the trend of the others, the blend continues with level 1 contributing effectively to conversion. Furthermore, Figure 3 shows that the blend with the lowest amount of Nile tilapia oil is the best blend for application under the conditions of this study.

From this perspective, Response Surface Methodology (RSM) uses statistical and mathematical techniques to provide the best ideal reaction conditions with the minimum possible runs of the experiment compared to a single-factor experimental design.^48,90^ It is noticeable that the contour surface graphs present the relationship of the three least significant factors related to the combination factor that had as a response to the biolubricant reaction yield.^84,85,87,88^

In this sense, it is clear that the relationship between the blend and molar ratio, which are the most relevant factors for research, show that at their minimum levels, they result in a higher reaction yield.^87,88^ According to Moreira et al. (2020), the molar ratio is a significant factor and often presents itself as a determining factor in the work carried out. Therefore, this is justified by the viscosity of the reaction medium, which increases as esterification progresses toward the production of the biolubricant. Faster agitation in the reaction medium can favor the mixing of substrates.^86^

Therefore, it is highlighted that in the lower and central left quadrants of all the graphs mentioned above, the best reaction yields are presented, that is, the best responses to the experiments carried out in the Taguchi planning.^84,85,87,88^ This arises from the impact of the factors combination and molar ratio on the reaction result, with time and catalyst having little effect compared to the others. This research is needed to evaluate the impacts of combination and molar ratio.^84,85,87,88,90^

Based on the analyses of the Taguchi method, a derivation was made to define an equation that delimits the conversion related to all the independent parameters analyzed in this work. This equation is described here as eq 5.

5 X⁡(%)=100.7−1.878A−0.409B−0.299C+0.04D

In eq 5, X is the conversion for the esterification reaction. A, B, C, and D are the encoded values of the molar ratio, the biocatalyst load, the blend, and the reaction time, respectively. The statistical analysis also concluded that the best time was presented in level 1, as 48 h (L1), the best blend was 20 % (L1), adequate biocatalyst load was defined as 10 % (L2), and finally, the molar ratio stood out in level 2, in the ratio of 1:5 (L2). Equation 5 indicates 97% conversion of the acids into esters, provided they are applied under the optimum conditions. However, the experimental application of this point resulted in 92.80 ± 0.03% actual conversion. Figure 4 shows the parity graph comparing the response predicted by eq 5 with the conversions defined in each design experiment.

**Figure 4:**
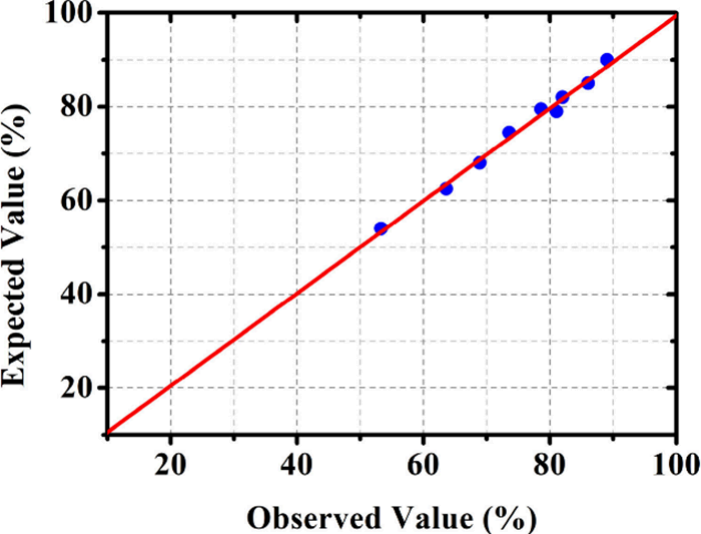
A regular probability graph was obtained to compare theoretical and experimental methods.

### Characterization of Product

In the ^1^H NMR spectrum (500 MHz, CDCl_3_) of the reaction medium, the signals for the formed octyl oleate can be observed. Despite several overlapping peaks in the spectrum of the reaction mixture, the most significant peaks can be identified to confirm the presence of these products in the reaction medium. Figure S1 (hydrogen NMR spectrum) highlights some essential chemical shifts of octyl oleate.^91^ Expansions, integration, and chemical shifts for the highlighted hydrogen atoms in the octyl oleate structure have been included to assign the signals to their respective chemical shift values and multiplicities.

The signal with a chemical shift near δ 5.48 was attributed to the hydrogen atoms on the sp2 carbon. When hydrogen atoms bonded to sp2 carbons are in the trans position, the peaks exhibit the splitting characteristics seen in Figure S1. This chemical profile, typical of alkenes, results in a signal multiplet at δ 5.48. The quartet signal at δ 4.11 corresponds to the hydrogen directly bonded to the oxygen of the ester group, reflecting its diversity and chemical shift.^92^ The methyl and methylene groups attached directly to the oxygen of the esters show these chemical changes due to the shielding effect of the electronegative oxygen atom on the neighboring hydrogen nuclei.

The peak at δ 3.52 was attributed to more diluted esters in the reaction medium due to their low intensity in the spectrum. The triplet observed at δ 2.70 is characteristic of methylene hydrogens in the α position to the carbonyl group of esters.^93^ The signals between δ 1.29 and δ 1.76 are related to the methyl and methylene groups common to esters in the reaction medium. Finally, the signal at δ 0.88 was attributed to the methyl group furthest from the functional group, showing more excellent shielding, lower chemical shift, and corresponding multiplicity.^93^

In Figure S2 (carbon spectrum NMR), the peaks for the 26 carbons in the structure of octyl oleate and their respective chemical shifts in ppm are highlighted. In the spectrum, the peak for the dehydrogenated carbon present in esters is near δ 175.0, confirming the formation of the ester and the consumption of the substrate. The number of carbons with the highest intensity was established as the primary structure, corresponding to the number of identical carbon nuclei for octyl oleate.^94^

This study used gas chromatography coupled with mass spectrometry (GC/MS) to define and quantify all the esters obtained from the esterification of fatty acids when catalyzed by ET2 lipase. This analysis aims to highlight the most essential esters obtained in the process. Thus, the composition of the fatty acid esters from the mixture of Nile tilapia oil and neem oil catalyzed by ET2 lipase was evaluated and discussed below.

GC/MS analysis highlighted the compounds 2-ethylhexyl ester, hexadecyl ester, octadecyl ester and octyl ester, which are essential in the formulation of biolubricants due to their chemical and physical properties. 2-Ethylhexyl ester is a compound responsible for ensuring high thermal stability and lubricity, so its presence in the composition of the biolubricant produced indicates resistance to high temperature and pressure conditions. Another characteristic of this ester is its resistance to oxidation, which extends the useful life of the biolubricant. The hexadecyl ester was also identified, which contributes by increasing fluidity and stability at low temperatures and, like the previous ester, ensures excellent lubricity. This greater resistance to low temperatures is essential for application in cold environments, guaranteeing a versatile application for this biolubricant.

Also determined in the GC/MS analysis were concentrations of octadecyl ester, a fundamental component for ensuring the biolubricant’s adequate viscosity, increasing its thickness and, therefore, ability to form a protective layer on metal surfaces. This characteristic is fundamental in reducing friction and wear on moving parts where the biolubricant is applied. Finally, the last ester highlighted in the analysis was octyl ester, commonly used in biolubricants because it allows for better solubility with oils. This characteristic makes the biolubricant more stable and has adequate lubricity. It also plays an important role when considering the need to reduce friction between contact surfaces, contributing to energy efficiency in mechanical systems.

Therefore, GC/MS analysis is necessary and efficient, as it highlights all the esters produced. These esters are essential for the biolubricant as they affect the main characteristics, ranging from friction reduction, thermal stability, and oxidation resistance to efficiency at low and high temperatures. With this, it is possible to consider that the biolubricant produced is an effective alternative to petroleum-based lubricants, with the added benefit of being more sustainable and biodegradable.

The characterization of the biolubricant produced from the mixture of Nile tilapia and neem oil was complemented by Fourier Transform Infrared Spectroscopy (FT-IR). This work applied FT-IR to highlight the bands characteristic of biolubricant materials, corroborating the experimental results already presented. Therefore, Figure 5 shows the bands highlighted in the material analyzed and their relationship with the leading bands in the analysis of biolubricants.

**Figure 5:**
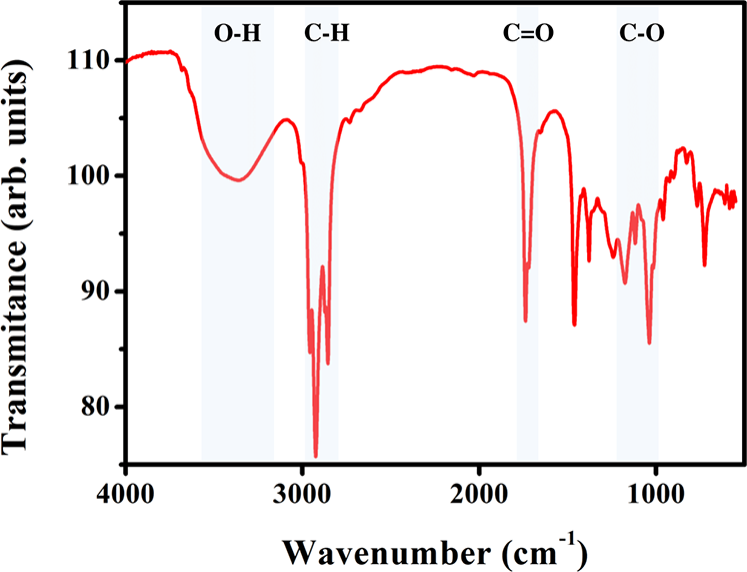
FT-IR spectra of the produced esters.

The FT-IR analysis highlighted the band at approximately 3,400 cm^–1^, representing hydroxyl groups (OH), usually related to water absorption or functional groups in the oil compounds. This band also highlights the free fatty acids in the oil blends used; however, the peak was downward in this band, indicating that the acids were consumed to synthesize biolubricants. Another prominent band is in the 2,900 cm^–1^ regions, generally associated with methyl and methylene groups, corresponding to the stretching vibrations of carbon–hydrogen (C–H) bonds present in aliphatic chains, typical of free fatty acid esters. These chains are related to the guarantee of viscosity, thermal stability, and resistance to oxidation, which are fundamental characteristics of biolubricants.

The band at approximately 1700 cm^–1^, representing the carboxylic groups (C=O), showed a sharp peak. This band highlights the esters produced during esterification, indicating the production of the biolubricant. The last band highlighted is in the region of approximately 1100 cm^–1^. This band also defines the ester groups of the material analyzed (C—O). As in the previous one, the peak indicates the effective production of biolubricant in this region. Therefore, by examining all the bands in the study, it is possible to define that the FT-IR analysis indicated the effective production of biolubricants from the blend of oils studied catalyzed by the ET2 lipase, corroborating the experimental results presented.

Viscosity and density for esters using esters from a mixture of neem oil and Nile tilapia oil in synthesize a biolubricant were analyzed. Higher kinematic viscosity benefits mechanical fuel pumps because it minimizes fuel leakage, enhancing injection pressure and mass of injected gasoline.^95^ Conversely, in compression ignition engines, reduced viscosity speeds up fuel spray atomization, makes fluid flow more accessible, and shortens ignition delay periods. For liquid fuels, viscosity—a measurement of a fluid’s resistance to tensile or shear stress—is essential.^96^ The kinematic viscosity exhibited a precise value of 2.4511 mm^2^/s at a temperature of 40 °C, as determined per the ASTM D 341 standard. This measurement categorizes the biolubricant within the ISO VG (Viscosity Grade) 3 classification. ISO VG 3 oils are characterized by their low viscosity, which makes them particularly suitable for applications in systems that operate under low load and low-speed conditions.

The ASTM D2270 and ISO 2909 standards only describe petroleum products with a kinematic viscosity greater than 2 mm^2^/s at 100 °C. Hence, the kinematic viscosity values of waste fish oil were not appropriate for computing the Viscosity Index (VI).^6,97,98^ The density of a material or liquid is defined as its mass per unit volume. The combination of neem and residual fish oil has a low density of 0.84259 g/cm^3^ at 40 °C, making it ideal for lubricating systems with light loads and slow speeds.

Sajeeb and Rajendrakumar (2019) proposed comparing the biolubricant properties obtained from coconut and mustard oil mixtures. The authors concluded that the B5 mixture presented superior properties to mineral oil.^99^ Kamarapu et al. (2022) investigated the tribological characteristics of palm oil and mineral oil mixtures. The authors obtained promising answers as the coefficient of friction was reduced.^100^ Given the studies, there is an emergence in the development of alternative routes for synthesizing and evaluating properties. The importance of developing mixtures of different generations of biofuel is noted in ensuring energy security.

### Study *in Silico*

Based on the literature^101^ and with some adaptations^102^ this molecular coupling simulation study was used to elucidate the hydrolysis interaction’s reaction between the blend of the residual oil of tilapia (*Oreochromis niloticus*) and neem (*Azadirachta indica*) and ET2 lipase for the possible formation of esters with bio-lubricant characteristics (Figure 6).

**Figure 6:**
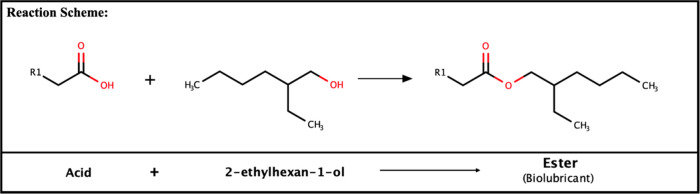
Reaction of the blend of the residual oil of tilapia (*Oreochromis niloticus*) and neem (*Azadirachta indica*) composition with 2-ethylhexan-1-ol alcohol catalyzed by the ET2 lipase.

The docking molecule by Autodock vina made it possible to list the affinity and RMSD energies of the ligands, as shown in Table 5. However, all are allocated in the same region of the protein’s active site, which is usually constituted by the catalytic triad (Ser-His-Asp), in the case of the ET2 Ser 153, His 268, and Asp 206.^64,65^

**Table 5:** Molecular Docking Result with Its Dock Scores

**Acid Name**	**PubChem**	**Energy Affinity** **(kcal/mol)**	**RMSD (Å)**
Linoleic acid	CID3931	–5.9	2.0
Octadecanoic acid	CID5281	–5.7	2.0
Oleic acid	CID445639	–5.5	1.8
Decanoic acid	CID2969	–5.5	2.0
Hexadecanoic acid	CID985	–5.3	2.0

NAC’s are defined as compatible with conformations to the catalytic site attack on the electrophilic carbon of the acyl group.^103^ In a typical NAC the distance between the oxygen from the Ser 153 (ET2) residue and the carbonyl carbon is usually observed to be close to 3 Å in length, and the same atoms, together with the carbonyl oxygen molecule, tend to form an angle of approximately 60°, with a maximum of 90°.^104^

In general, the carboxylic acids evaluated as ligands in this study presented a favorable approximation to the enzyme’s catalytic site. This shows that the interaction energies between the substrates and the residues of the catalytic triad and neighboring residues are favorable. However, among the ligands evaluated, octanoic acid (B), oleic acid (C), decanoic acid (D), and hecadecanoic acid presented NAC-type interactions with Ser 153. The distances of the interactions are shown in Table 6. In contrast, linoleic acid (A) has not showed NAC-type interactions with any of the residues of the catalytic triad but presented a strong interaction via hydrogen bond with the Tyr 29 residue.^105^ This indicates that, although the ligand does not establish direct interactions with the catalytic triad, its allocation within the catalytic site is favored by the neighboring residues.^104^ The expansion of hydrophobic interactions near residues Ser 153, His 268, and Asp 206 are essential for stabilizing and accommodating these ligands in the enzyme active site. These hydrophobic regions of the substrates tend to interact with hydrophobic pockets in the enzyme active site, where they contribute to correctly positioning and orienting the molecule in the catalytic cavity. Another contribution of these hydrophobic interactions is that with the increase in these interactions, there is a decrease in the number of water molecules between the substrate and the active site, which leads to the rise in the global entropy of the system, thermodynamically favoring this stabilization.^106^ Thus, the greater the number of interactions the ligand performs with the amino acid residues, the greater the stability of the enzyme complex formed, keeping them closer to the region where catalysis occurs for longer.^107^

**Table 6:** Interactions of Ligands with Lipase after Docking

	**Waste Involved**	
**Substrate**	**Hydrogen Bonds**	**Hydrophobic Interactions/Salt Bridge**
Linoleic acid (A)	Tyr 29 (3.80 Å)	Tyr 92, Ile 94, Arg 95, Val 98, Leu 262, Phe 265, His 268 (CT), Asp 276
Octadecanoic acid (B)	Tyr 29 (3.50 Å)	Tyr 92, Asn 232, Phe 265, His 266, His 268 (CT), Val 269, Trp 270, Leu 283, Leu 285
	Ser 153 (3.00 Å) (NAC)	
Oleic acid (C)	Tyr 29 (2.80 Å)	Tyr 92, Ile 94, Phe 265, His 268 (NAC), Val 269, Leu 283, Leu 285
	Ser 153 (3.30 Å) (NAC)	
Decanoic acid (D)	Gly 90 (3.00 Å)/(3.90 Å)	Tyr 29, Ser 91, Tyr 92, Ile 94
	Ser 153 (3.10 Å) (NAC)	
	Gly 155 (2.80 Å)	
Hexadecanoic acid (E)	Tyr 29 (2.90 Å)	Tyr 92, Ile 94, Leu 262, Leu 263, Phe 265, His 266, Val 269, Leu 283, Leu 285
	His 152 (3.30 Å)	
	Ser 153 (3.60 Å) (NAC)	
	His 268 (3.60 Å) (CT)	

In summary, the stability of the enzyme–substrate complexes appears to be directly influenced by the number and nature of molecular interactions established within the active site. Among the evaluated substrates, octadecanoic acid (B) exhibited the highest stability, supported by two well-positioned hydrogen bonds (Tyr 29 and Ser 153) and extensive hydrophobic interactions with residues such as Trp 270, Phe 265, and several leucines (Leu 283, Leu 285), which likely contribute to a strong binding affinity through hydrophobic packing. Similarly, hexadecanoic acid (E) demonstrated a highly stable profile, presenting four hydrogen bonds, two of them within favorable distances and numerous hydrophobic contacts with aliphatic and aromatic residues.^108^ Oleic acid (C) also formed a short hydrogen bond with Tyr 29 and hydrophobic contacts comparable to hexadecanoic acid (E), though with slightly fewer interacting residues, possibly leading to a marginally lower stability. Linoleic acid (A), despite its relatively long carbon chain, established only one hydrogen bond at a longer distance (3.80 Å), with moderate hydrophobic interactions, suggesting reduced stabilization.

In contrast, decanoic acid (D), the shortest chain in the series, displayed three hydrogen bonds but limited hydrophobic interactions, which may result in weaker binding due to the lower surface contact area. These observations reinforce the critical role of hydrophobic interactions, particularly those involving bulky residues in stabilizing long-chain fatty acids within the active site, and suggest that an optimal combination of hydrogen bonding and hydrophobic contact is essential for high-affinity binding.^109^ In particular, the hydrophobic interactions between carboxylic acids and residues Ile 94 and Leu 154 have been crucial for the enzymatic mechanism of conversion of these acids into esters.^56^ All residues interacting with each ligand and the type of interaction are shown in Table 6 and Figure 7.

**Figure 7:**
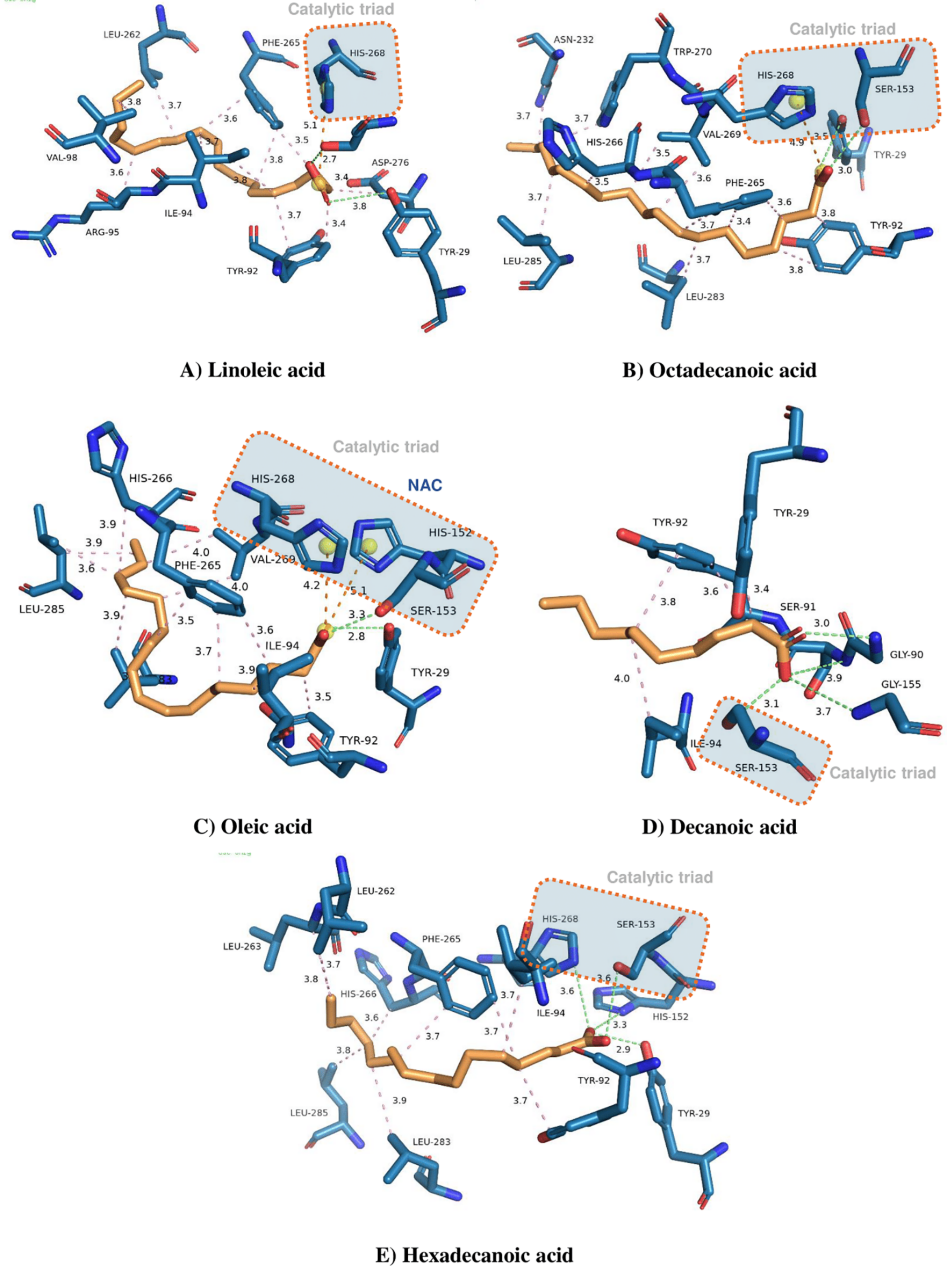
Active site of ET2 lipase with the catalytic triad Ser 153, His 268, and Asp 206 (A–E).

### Molecular Dynamics Simulations

#### Root Mean Square Deviation (RMSD)

It was observed that the ligands studied were correctly accommodated in the catalytic site of ET2 lipase.^110^ Thus, through the lipase–ligand complexes (Figure 8), simulation studies were carried out to evaluate not only the conformational changes of the enzyme but also its stability after each conformational change. The Root Mean Square Deviation (RMSD) of the lipase–ligand complexes was used to evaluate the extent to which conformational changes occurred in the studied molecule during the simulation. Figure 8 shows the RMSD behaviours of the complexes studied in the equilibration stage.

**Figure 8:**
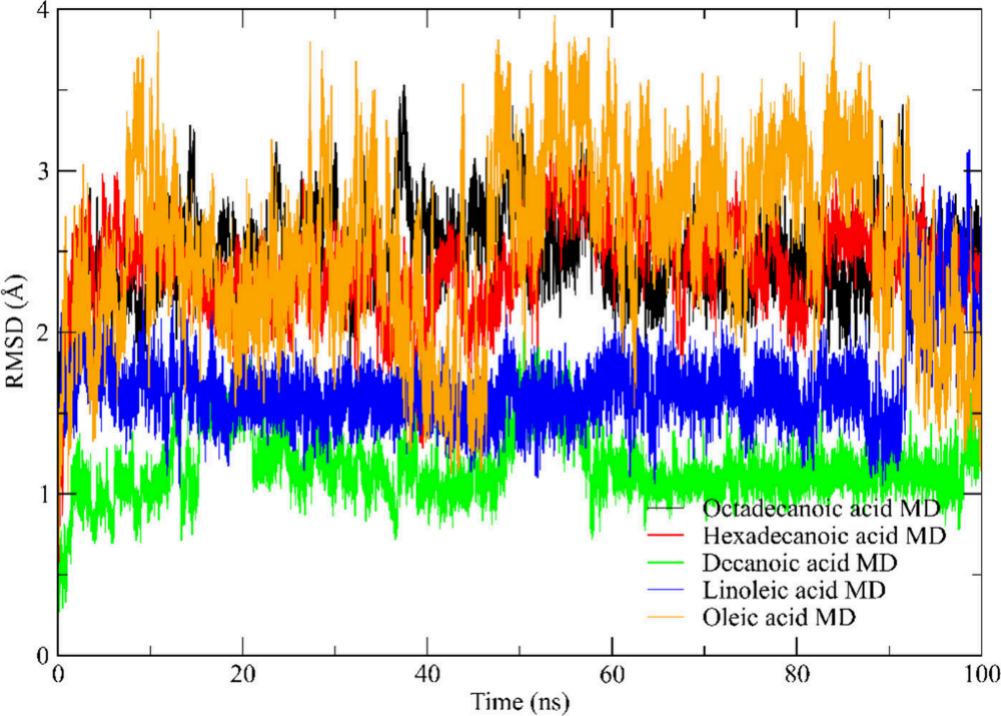
Root Mean Square Deviation (RMSD) for the initial conformation of the complexes versus the simulation time (nanoseconds) in the production step. Octadecanoic acid (black); Hexadecanoic acid (red); Decanoic acid (green); Linoleic acid (blue); and Oleic acid (orange).

From the equilibrium simulations of the lipase–ligand complexes in the solvent, it was possible to obtain preliminary information on the behavior of the conformations for the dynamics. In this step, it was observed that the RMSD stabilization values of the studied ligands oscillated between 1.0 and 3.2 Å in the evaluated time. These low RMSD values may be associated with the movement of ions and solvents in the system during the initial conformation of the complexes. Figure 8 shows the results obtained in the production stage.

Decanoic and linoleic acid showed RMSD values below 2 Å and presented stable complexes during the simulation. The remaining carboxylic acids showed stability but with RMSD above 2.0 Å. Thus, the theoretical and experimental results presented in this work indicate that blend oil compounds form stable complexes, which may indicate a viable alternative for future applications in the production of biolubricants.

#### Hydrogen Bonds

Intermolecular hydrogen bonds, a cornerstone of enzyme–ligand complex stability, have been a long-standing focus of biochemical research. However, in a recent study by Ragunathan et al. (2018),^111^ we delve deeper into these bonds, specifically in the context of lipase–ligand complexes, revealing novel insights through molecular dynamics simulations.

The analysis primarily focused on examining the formation of intermolecular hydrogen bonds between the ligands and lipase throughout both equilibration and production phases. Figure 9 from the study depicted the fluctuations in hydrogen bond networks during these stages, shedding light on the variations in bond formation.

**Figure 9:**
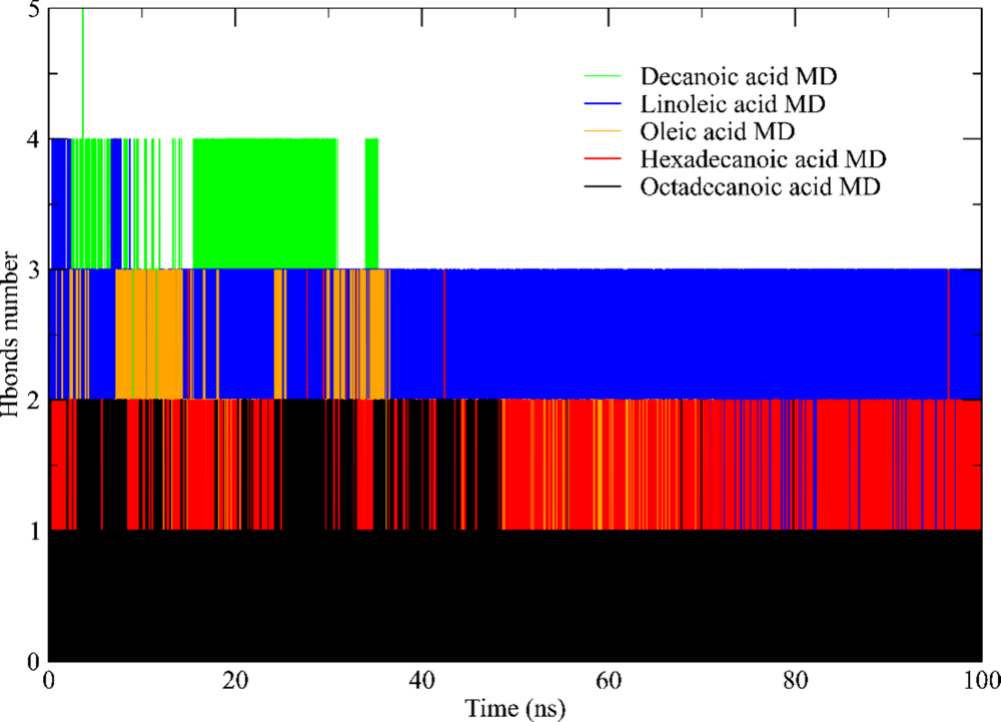
Hydrogen bonds formed between the protein and the ligand during the two simulation steps.

A notable discovery highlighted the dynamic formation of hydrogen bonds, with the bond count fluctuating between 1 and 5 throughout the simulation period. The observation regarding decanoic acid and linoleic acid was fascinating, as it demonstrated the formation of up to 5 and 3 hydrogen bonds during the 100 ns trajectory, respectively.

The oleic acid has formed networks of bonds. The disruption of hydrogen bonds indicated shifts in stability, often attributed to interactions such as van der Waals or hydrophobic forces, as Qin et al. emphasized in 2021.^108^ Additionally, the analysis revealed a direct correlation between the length of the ligands’ carbon chains and the average bond count, reinforcing insights from previous molecular docking investigations.^112^

A key revelation from this examination was the differentiation between dynamic (molecular dynamics) and static (molecular docking) processes, as elucidated by Fatma et al. (2021).^113^ While molecular docking provides valuable insights into static interactions, molecular dynamics simulations capture the dynamic evolution of these complexes, offering a more comprehensive understanding of their stability.

Our investigation into the dynamic interplay of intermolecular hydrogen bonds in lipase–ligand complexes deepens our understanding of protein–ligand recognition processes. It holds significant implications for drug design and enzyme engineering. These findings underscore the practical relevance and impact of our research.

#### RMSF Analysis

We conducted an RMSF analysis of the system to delve into the dynamics and stability of each protein residue throughout the 100 ns simulation trajectory. Figure 10 showcases the primary interactions within the investigated leading complexes, particularly those composed of blend oil. The findings hint at substantial conformational shifts in the compound–ET2 lipase complexes during the simulation period.

**Figure 10:**
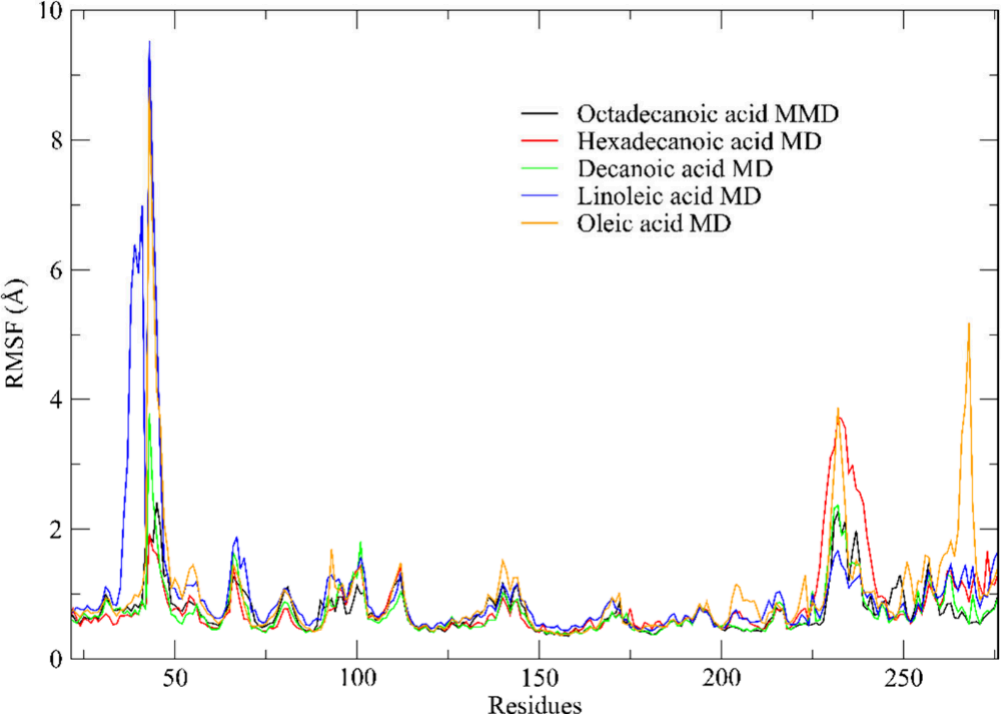
Root Mean Square Fluctuation (RMSF), concerning the initial confirmation of the ligand–enzyme complex versus the simulation time (ns) in the production simulation step of the MD with blend oil composition/ET2 lipase.

Our results unveil consistent fluctuations in the molecular dynamics simulation trajectories across all complexes, showcasing notable correlations with key replication residues, as highlighted in studies by Qin et al. (2021)^108^ and Roe et al. (2020).^114^ Notably, all complexes formed between carboxylic acids and ET2 exhibited RMSF values higher than 2.0 Å for Gly 47-Asn 48 and Asp 237 residues. However, an additional detail was observed that only oleic acid presented a value above 2.0 Å for His 268, which is part of the catalytic triad.

Despite the observed fluctuations, our results indicate satisfactory stability of the structures within an aqueous solution. The conformations derived from MD simulations, complexed with various ligands through docking techniques, offer crucial insights into the binding modes of small molecules across different enzyme folding states, as illuminated by.^115^

#### SASA Calculations

Our research, which combines the powerful computational tool of molecular dynamics (MD) simulations with the insightful metric of Solvent Accessible Surface Area (SASA), offers a unique perspective on the behavior of proteins in solution. Throughout 100 ns, we applied this novel approach to closely monitor the SASA of complexes formed with fish oil compositions, unveiling intricate dynamics and stability patterns within biomolecules.^116^

Our SASA analysis produced intriguing trends. Notably, the SASA value for all carboxylic acids exhibited a marked increase during the simulation, indicative of structural relaxation^117,118^ (see Figure 11).

**Figure 11:**
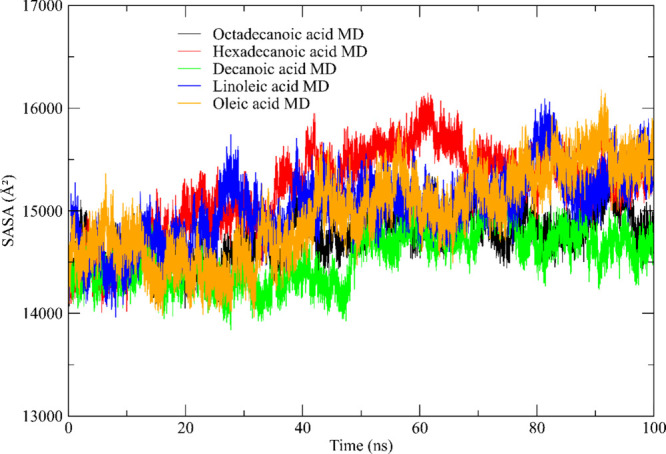
Solvent accessible surface area (SASA) of the ET2 lipase as a function of time from the MD simulations. The curves are running raw data averages with a window of 100 ns.

The negligible impact of ligand binding on SASA values was fascinating, implying that ligand–protein interactions did not significantly alter solvent accessibility to the protein surface.^119^ Following 100 ns of simulation, SASA values stabilized around a constant, suggesting equilibrated systems were sampled. However, interestingly, the complexes containing stabilizing monovalent ions exhibited the highest SASA values, although higher concentrations of these ions correlated with smaller areas, potentially indicating a compaction of the protein structures induced by the surface charge.

In conclusion, our SASA analysis during MD simulations has provided crucial insights into the dynamics and stability of fish oil composition complexes. These findings deepen our understanding of protein behavior and hold promise for guiding the design of compounds with enhanced interactions with protein surfaces or optimizing conditions for protein complex formation, opening up new avenues for future research and applications.

## Conclusions

This study investigated the efficiency of the Nile tilapia and neem oil blends, catalyzed by ET2 lipase, in synthesizing biolubricants. The statistical analysis concluded that among the parameters analyzed, the percentage of the Blend was the most significant. The point of highest conversion was defined as a molar ratio of 1:5 (L2), 10% biocatalyst loading (L2), 20% Nile tilapia oil blends (L1), and 48 h time (L1), indicating 97% conversion of acids into esters. Still, the experimental application of this point resulted in 92.80 ± 0.03% actual conversion.

GC/MS analysis efficiently highlights the esters produced, which are crucial for the biolubricant’s properties, such as friction reduction, thermal stability, and oxidation resistance. These characteristics make the biolubricant a viable, sustainable alternative to petroleum-based lubricants. The FT-IR analysis confirms the successful production of biolubricants from the blend of oils catalyzed by ET2 lipase, supporting the experimental results and indicating effective ester formation.

In conclusion, the comprehensive study of lipase ET2–ligand complexes via molecular dynamics (MD) simulations and various analytical metrics—such as RMSD, hydrogen bond formation, RMSF, and SASA—provided critical insights into the conformational stability and behavior of the enzyme–ligand systems. The RMSD analysis demonstrated that the ligands, particularly decanoic and linoleic acids, formed stable complexes, with low RMSD values indicating minor conformational changes during simulations. Hydrogen bond analysis revealed dynamic fluctuations in bond formation, which correlated with ligand carbon chain lengths, providing a deeper understanding of enzyme–ligand recognition processes. The RMSF analysis highlighted specific protein residues exhibiting significant conformational shifts. At the same time, the SASA calculations suggested that ligand binding had minimal impact on protein surface accessibility, yet higher ion concentrations influenced protein structure compaction. These findings contribute significantly to the fields of drug design, enzyme engineering, and the development of biolubricants, underscoring the practical relevance of MD simulations in understanding complex biochemical interactions.

## Supplementary Material



## Notes

Figure S1 showing the hydrogen nuclear magnetic resonance (^1^H NMR) spectrum of the ester mixture, with expansions highlighting octyl oleate as the main product, and Figure S2 showing the Carbon-13 Nuclear Magnetic Resonance (^13^C NMR) spectrum for octyl oleate, including an expansion of the carbonyl carbon region, confirming the formation of the ester (PDF)
