# Supplementary material for: Enzymatic Conversion of Mixed Neem (Azadirachta indica) and Nile Tilapia (Oreochromis niloticus) Oils into Biolubricants: A Green Biocatalytic Approach
Source: ACS Omega. 2025 May 20;10(21):21361–76. doi: 10.1021/acsomega.4c11707 (PMC12138628; doi:10.1021/acsomega.4c11707)
Supplement: Supplementary file 1 [file ao4c11707_si_002.pdf]

Supporting Information

Enzymatic Conversion of Mixed Neem (*Azadirachta indica*) and Nile Tilapia (*Oreochromis niloticus*) Oils into Biolubricants: A Green Biocatalytic Approach

Francisco Simão Neto[1], Patrick da Silva Sousa[1], Rafael Leandro Fernandes Melo[2], Antônio Luthierre Gama Cavalcante[3], Paulo Gonçalves de Sousa Junior[3], Sara Jessica Marciano[3], Diego Lomonaco[3], Raniere Dantas Valença[4], Frederico Ribeiro do Carmo[5], Marcos Carlos de Mattos[6], Maria Alexsandra de Sousa Rios[7], Paulo Roni de Souza[8], Aluísio Marques da Fonseca[8], Ada Sanders Lopes[8], e José Cleiton Sousa dos Santos[8*]

[1]Departamento de Engenharia Química, Universidade Federal do Ceará, Fortaleza, CE 60455-760, Brasil; fcosimao@aluno.unilab.edu.br ; patrick@aluno.unilab.edu.br

[2]Departamento de Engenharia Metalúrgica e de Materiais, Universidade Federal do Ceará−UFC, Campus do Pici, Fortaleza, 60714-903 Ceará, Brasil; rafael.melo@ifce.edu.br

[3]Departamento de Química Orgânica e Inorgânica, Universidade Federal do Ceará, Fortaleza, CE 60455-760, Brasil. lumonaco@ufc.br ; jessicamsara@gmail.com; luthi2011@gmail.com; paulogdsj@gmail.com

[4]Universidade Federal Rural do Semi-Árido, Mossoró, RN, 52625-900, Brasil. raniere.valenca@ufersa.edu.br

[5]Núcleo de Pesquisa em Economia de Baixo Carbono, Centro de Engenharias, Universidade Federal Rural do Semi-Árido, Mossoró, RN, 52625-900, Brasil. frederico.ribeiro.c@ufersa.edu.br

[6]Departamento de Química Analítica e Físico-química, Universidade Federal do Ceará, Fortaleza, CE 60455-760, Brasil. mcdmatto@ufc.br

[7]Departamento de Engenharia Mecânica, Universidade Federal do Ceará, Fortaleza, CE 60455-760, Brasil; alexsandrarios@ufc.br

[8]Instituto de Engenharia e Desenvolvimento Sustentável, Universidade da Integração Internacional da Lusofonia Afro-Brasileira, Campus das Auroras, Redenção, CE 62790-970, Brasil; ada@unilab.edu.br; ronistil@hotmail.com ; aluisiomf@unilab.edu.br ; jcs@unilab.edu.br *

***Corresponding author:**

Prof. Dr. José Cleiton Sousa dos Santos
Institute of Engineering and Sustainable Development
University of International Integration of Afro-Brazilian Lusophony
Redenção, CE, Brazil, Zip-Code: 62790970
Phone: + 55 (85) 3332.6109
e-mail: jcs@unilab.edu.br (J.C.S.S.)

Additional information on the in silico study

Molecular docking was performed by AutoDock Vina [1] employing 3-ways multithreading, Lamarkian Genetic was performed. For docking of ET2 main protease complex, following parameters were used: number grid points in xyz (30 30 30), spacing (0.642), grid center in xyz (-34.282188 24.937188 73.691625). Other parameters were set to default. Input ligands with polar hydrogens were used in .pdbqt format. Between ten and forty molecular docking executions were performed, and several simulations were repeated in the same region of the biological receptor. Thus, to validate the simulation's performance and quantify the dockings' quality, the RMSD (root mean square deviation) scoring criterion was adopted, which suggests that a successful docking exhibits an RMSD value rmsd $\leq$ 2.0 Å [2]. The simulation data with the main receptor-ligand interactions were visualized by Discovery Studio software [3].

Molecular dynamics (MD) simulations were performed with the NAMD program [4]. The best conformations obtained in molecular coupling were solved in water in the TIP3P model [5], in the CHARMM36 force field, and in ions to neutralize the total system load. Finally, it was submitted for energy minimization using the Steepest Descent method. The system was then introduced to NVT and NPT balances under conditions described by Langevin [6]. The system production simulations were performed with a time of 100 ns.

The quality of the structures obtained in MDs was evaluated using the following parameters with NAMD:

- Potential Energy (kcal/mol) [7];
- Protein-Ligand Interaction Energy (kcal/mol);
- The mean quadratic deviation of the atomic positions of proteins, binders, and distances between them (RMSD, Å), and the mean quadratic deviation of the nuclear positions of proteins, ligands, and distances between them (RMSD, Å);
- Hydrogen bonds were evaluated with Visual Molecular Dynamics (VMD) [8].
- The mean quadratic fluctuation of the minimum distances between proteins and ligands was observed in MD (RMSF, Å) [9]. The graphs were generated using the Qtrace program [10].

Support figures

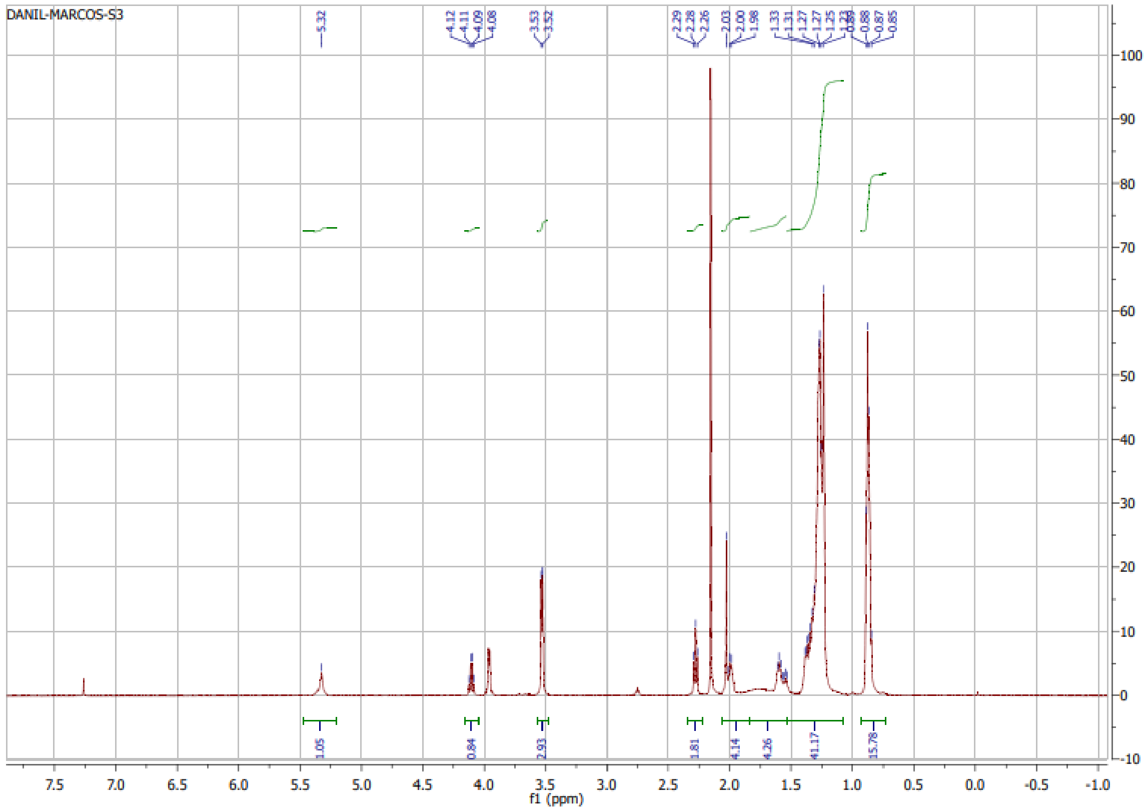

Figure S1: ^{1}H Nuclear Magnetic Resonance (NMR) spectrum for the sample of ester mixture with expansions of octyl oleate as the major product.

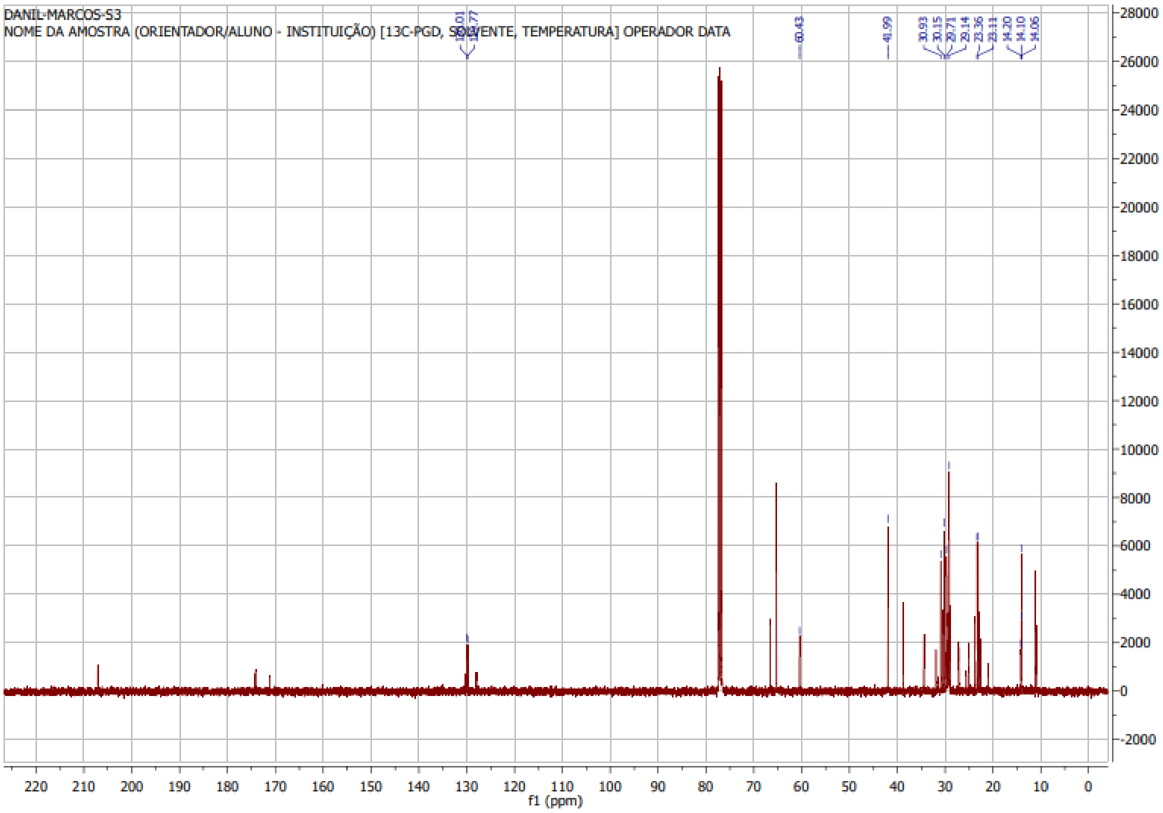

Figure S2: Carbon-13 Nuclear Magnetic Resonance (^{13}C NMR) spectrum for octyl oleate with an expansion for the carbonyl carbon, confirming the formation of the ester.

References

(1) Trott, O.; Olson, A. J. AutoDock Vina: Improving the Speed and Accuracy of Docking with a New Scoring Function, Efficient Optimization, and Multithreading. *J Comput Chem* **2010**, *31* (2), 455–461. https://doi.org/10.1002/jcc.21334.

(2) Sutherland, J. J.; Nandigam, R. K.; Erickson, J. A.; Vieth, M. Lessons in Molecular Recognition. 2. Assessing and Improving Cross-Docking Accuracy. *J Chem Inf Model* **2007**, *47* (6), 2293–2302. https://doi.org/10.1021/ci700253h.

(3) Biovia. Dassault Systemes BIOVIA, Discovery Studio Modelling Environment, Release 4.5. *Accelrys Software Inc.* San Diego 2015.

(4) Phillips, J. C.; Braun, R.; Wang, W.; Gumbart, J.; Tajkhorshid, E.; Villa, E.; Chipot, C.; Skeel, R. D.; Kalé, L.; Schulten, K. Scalable Molecular Dynamics with NAMD. *J Comput Chem* **2005**, *26* (16), 1781–1802. https://doi.org/10.1002/jcc.20289.

(5) Kato, K.; Nakayoshi, T.; Kurimoto, E.; Oda, A. Molecular Dynamics Simulations for the Protein–Ligand Complex Structures Obtained by Computational Docking Studies Using Implicit or Explicit Solvents. *Chem Phys Lett* **2021**, *781* (139022). https://doi.org/10.1016/j.cplett.2021.139022.

(6) Farago, O. Langevin Thermostat for Robust Configurational and Kinetic Sampling. *Physica A: Statistical Mechanics and its Applications* **2019**, *534* (122210). https://doi.org/10.1016/j.physa.2019.122210.

(7) Diez, M.; Petuya, V.; Martínez-Cruz, L. A.; Hernández, A. Insights into Mechanism Kinematics for Protein Motion Simulation. *BMC Bioinformatics* **2014**, *15* (1). https://doi.org/10.1186/1471-2105-15-184.

(8) Humphrey, W.; Dalke, A.; Schulten, K. VMD: Visual Molecular Dynamics. *J Mol Graph* **1996**, *14* (1), 33–38. https://doi.org/10.1016/0263-7855(96)00018-5.

(9) Arshia, A. H.; Shadravan, S.; Solhjoo, A.; Sakhteman, A.; Sami, A. De Novo Design of Novel Protease Inhibitor Candidates in the Treatment of SARS-CoV-2 Using Deep Learning, Docking, and Molecular Dynamic Simulations. *Comput Biol Med* **2021**, *139*. https://doi.org/10.1016/j.compbiomed.2021.104967.

(10) Lima, A. H.; Souza, P. R. M.; Alencar, N.; Lameira, J.; Govender, T.; Kruger, H. G.; Maguire, G. E. M.; Alves, C. N. Molecular Modeling of T. Rangeli, T. Brucei Gambiense, and T. Evansi Sialidases in Complex with the DANA Inhibitor. *Chem Biol Drug Des* **2012**, *80* (1), 114–120. https://doi.org/10.1111/j.1747-0285.2012.01380.x.
